# Supplementary material for: Could early tweet counts predict later citation counts? A gender study in Life Sciences and Biomedicine (2014–2016)
Source: PLoS One. 2020 Nov 2;15(11):e0241723. doi: 10.1371/journal.pone.0241723 (PMC7605688; doi:10.1371/journal.pone.0241723)
Supplement: S1 Appendix — (DOCX) [file pone.0241723.s001.docx]

| **Variables** | **VIF** | **CVIF K** |
| --- | --- | --- |
| Title length | 1.1431 | 0 |
| Gender (first author) | 1.0285 | 0 |
| Gender (last author) | 1.0266 | 0 |
| Mega Journal | 1.0144 | 0 |
| Number of MeSH topics | 1.1302 | 0 |
| MeSH-Anatomy | 1.1326 | 0 |
| MeSH-organism | 1.1719 | 0 |
| MeSH-Diseases | 1.1176 | 0 |
| MeSH-Chemicals and drugs | 1.1214 | 0 |
| MeSH- Analytical. Diagnostic and Therapeutic Techniques and Equipment | 1.2800 | 0 |
| MeSH-Psychiatry and Psychology | 1.0296 | 0 |
| MeSH-Health care | 1.0613 | 0 |
| SNIP | 1.2224 | 0 |
| OA status | 1.2774 | 0 |
| Number of authors | 1.6446 | 0 |
| Number of countries | 2.0221 | 0 |
| Lay summary | 1.0070 | 0 |
| F1000 score | 1.0712 | 0 |
| Funding | 1.2858 | 0 |
| Paper length | 1.0017 | 0 |
| Abstract readability | 1.0294 | 0 |
| First author-Number of publications | 2.9286 | 0 |
| First author- Number of citations | 2.0466 | 0 |
| First author- Number of self-citations | 2.7413 | 0 |
| Time | 1.0173 | 0 |
| Totla number of citations | 1.0996 | 0 |
| Last author- Number of publications | 2.6795 | 0 |
| Last author- Number of citations | 2.1414 | 0 |
| Last author- Number of self-citations | 2.3816 | 0 |

1 --> COLLINEARITY is detected by the test

0 --> COLLINEARITY is not detected by the test
