# Supplementary material for: Could early tweet counts predict later citation counts? A gender study in Life Sciences and Biomedicine (2014–2016)
Source: PLoS One. 2020 Nov 2;15(11):e0241723. doi: 10.1371/journal.pone.0241723 (PMC7605688; doi:10.1371/journal.pone.0241723)
Supplement: S2 Appendix — (DOCX) [file pone.0241723.s002.docx]

| **Variables** | **VIF** | **CVIF K** |
| --- | --- | --- |
| Title length | 1,14 | 0 |
| Gender (first author) | 1,03 | 0 |
| Gender (last author) | 1,03 | 0 |
| Mega Journal | 1,01 | 0 |
| Number of MeSH topics | 1,13 | 0 |
| MeSH-Anatomy | 1,13 | 0 |
| MeSH-organism | 1,17 | 0 |
| MeSH-Diseases | 1,12 | 0 |
| MeSH-Chemicals and drugs | 1,12 | 0 |
| MeSH-Analytical. Diagnostic and Therapeutic Techniques and Equipment | 1,28 | 0 |
| MeSH-Psychiatry and Psychology | 1,03 | 0 |
| MeSH-Health care | 1,06 | 0 |
| SNIP | 1,31 | 0 |
| OA status | 1,28 | 0 |
| Number of authors | 1,64 | 0 |
| Number of countries | 2,02 | 0 |
| Lay summary | 1,01 | 0 |
| F1000 score | 1,08 | 0 |
| Funding | 1,29 | 0 |
| Paper length | 1,00 | 0 |
| Abstract readability | 1,03 | 0 |
| First author-Number of publications | 2,82 | 0 |
| First author- Number of citations | 2,93 | 0 |
| First author- Number of self-citations | 2,05 | 0 |
| Early tweet counts | 2,74 | 0 |
| Later citation counts | 1,25 | 0 |
| Time | 1,09 | 0 |
| Last author- Number of publications | 1,09 | 0 |
| Last author- Number of citations | 2,15 | 0 |
| Last author- Number of self-citations | 2,38 | 0 |

1 --> COLLINEARITY is detected by the test

0 --> COLLINEARITY is not detected by the test
